# Ecological aspects of Phlebotomines (Diptera: Psychodidae) and the transmission of American cutaneous leishmaniasis agents in an Amazonian/ Guianan bordering area

**DOI:** 10.1186/s13071-018-3190-0

**Published:** 2018-11-29

**Authors:** Thiago Vasconcelos dos Santos, Ghislaine Prévot, Marine Ginouvès, Rosemere Duarte, Fernando Tobias Silveira, Marinete Marins Póvoa, Elizabeth Ferreira Rangel

**Affiliations:** 1 Programa de Pós Graduação em Biologia de Agentes Infecciosos e Parasitários, Instituto de Ciências Biológicas, Universidade Federal do Pará, Belém, Pará State Brazil; 2 Seção de Parasitologia, Instituto Evandro Chagas (Secretaria de Vigilância em Saúde, Ministério da Saúde), Ananindeua, Pará State Brazil; 3 Département de Médecine, Ecosystemes Amazoniens et Pathologie Tropicale, EA 3593, Labex CEBA, Université de Guyane, Cayenne, French Guiana; 4 Laboratório de Imunodiagnóstico, Escola Nacional de Saúde Publica Sérgio Arouca, Fundação Oswaldo Cruz, Rio de Janeiro, Rio de Janeiro State Brazil; 5 Laboratório Interdisciplinar de Vigilância Entomológica em Diptera e Hemiptera, Instituto Oswaldo Cruz/ Fundação Oswaldo Cruz, Rio de Janeiro, Rio de Janeiro State Brazil

**Keywords:** *Leishmania guyanensis*, Disease transmission, Vector ecology, State of Amapá, Brazil

## Abstract

**Background:**

An entomological study was conducted in the municipality of Oiapoque (lower Oyapock River Basin) in the Brazilian side bordering French Guiana to gain information on the transmission pattern of American cutaneous leishmaniasis (ACL) in that region, presumed to reflect the classical Amazonian/Guianan enzootic scenario.

**Methods:**

Three ecologically isolated forested areas near urban environments were surveyed during the rainy and dry seasons of 2015 and 2016, using a multi-trapping approach comprising ground-level and canopy light traps, black and white colored cloth Shannon traps and manual aspiration on tree bases. Female phlebotomines were dissected to find infections and isolate flagellates from *Leishmania* spp. The strains were characterized by restriction fragment length polymorphism analysis and compared with those of local ACL cases and World Health Organization reference strains.

**Results:**

*Nyssomyia umbratilis*, *Trichopygomyia trichopyga* and *Evandromyia infraspinosa* were the most frequently found species. Findings on relative abundance, spatiotemporal vector/ACL congruence, natural infections and anthropophilic insights strengthened the Guianan classical transmission of *Leishmania* (*Viannia*) *guyanensis* by *Ny. umbratilis* and suggested further investigations for *Ev. infraspinosa*. *Nyssomyia umbratilis* showed an eclectic feeding habit, including bird blood. Ecological data and literature reports also included *Psychodopygus squamiventris maripaensis* and *Bichromomyia flaviscutellata* on the list of suspected vectors.

**Conclusions:**

These findings contributed to understanding ACL ecoepidemiology in the Amazonian/Guianan scenario. Local studies are required to better comprehend the *Leishmania* spp. enzootic mosaic in specific ecotopes.

## Background

Phlebotomine sand flies (Diptera: Psychodidae) play determinant roles in transmitting Leishmaniinae (Kinetoplastida: Trypanosomatidae) parasites, the causative agents of leishmaniasis [1–5]. In American cutaneous leishmaniasis (ACL), biologically compatible vector/parasite/reservoir arrangements can be driven naturally or triggered by ecological/human-made pressures, resulting in highly diverse and countless natural transmission cycles [2, 6–8]. Such diversity is reflected in the emergence of a wide and worrisome clinical-immunological spectrum, since some phlebotomine species can carry ACL agents that cause life-threatening and debilitating disease forms such as the anergic diffuse and mucosal forms [9].

In the Amazonian/Guianan region, the major *Leishmania*/vector-recognized transmission cycle involves *Leishmania* (*Viannia*) *guyanensis* and the phlebotomine *Nyssomyia umbratilis* [10–13]. However, in this region, an emerging ACL pattern is currently being considered, and at least four dermotropic *Leishmania* species have been reported: *L.* (*V.*) *braziliensis*, *L.* (*Leishmania*) *amazonensis*, *L.* (*V*.) *lainsoni* and *L.* (*V.*) *naiffi* [14]. Within that region, in the Oyapock River Basin, *L.* (*V.*) *guyanensis* accounts for 81% of ACL etiology, followed by *L.* (*V*.) *braziliensis* (17%) and *L.* (*V.*) *lainsoni* (2%) [15]. However, in this region, ACL foci are assumed to be concentrated in the upper basin, where gold mining represents a high-risk factor for exposure [16]. Underreported outbreaks associated with periurban forested environments should be surveyed.

The present study assessed potential ACL transmission cycles in the lower Oyapock River Basin to promote knowledge on phlebotomine ecology, mainly focusing on species composition, multi-trapping stratification, blood-source investigation and natural *Leishmania* spp. infections. The isolates obtained were also compared with human isolates.

## Methods

### Study area

The municipality of Oiapoque (03°49'29"N, 51°49'05"W) is in the Oyapock River Basin, a border region between Brazil and the Ultramarine Department of French Guiana. It is the northernmost municipality of the Brazilian State of Amapá (AP) and is limited by the AP municipalities of Calçoene, Serra do Navio and Pedra Branca do Amapari to the south, by Laranjal do Jari to the west, by the Atlantic Ocean to the east, and by the French-Guianan communes of Camopi and Saint Georges de l’Oyapock to the north. Oiapoque has a population of approximately 24,263 distributed over 22,625 km^2^ [17]. During 2008–2017, a total of 1299 new ACL cases were registered by the health services in Oiapoque (average of 118 cases/year), with 560 shown to be autochthonous for that municipality (average of 50 cases/year). Because of Oiapoque’s border characteristics, ACL epidemiology is a mosaic of “binational” infections, as half of ACL-notified cases were autochthonous from Brazil and half were likely imported from French Guiana [18].

### Sampling sites

**Fig. 1:**
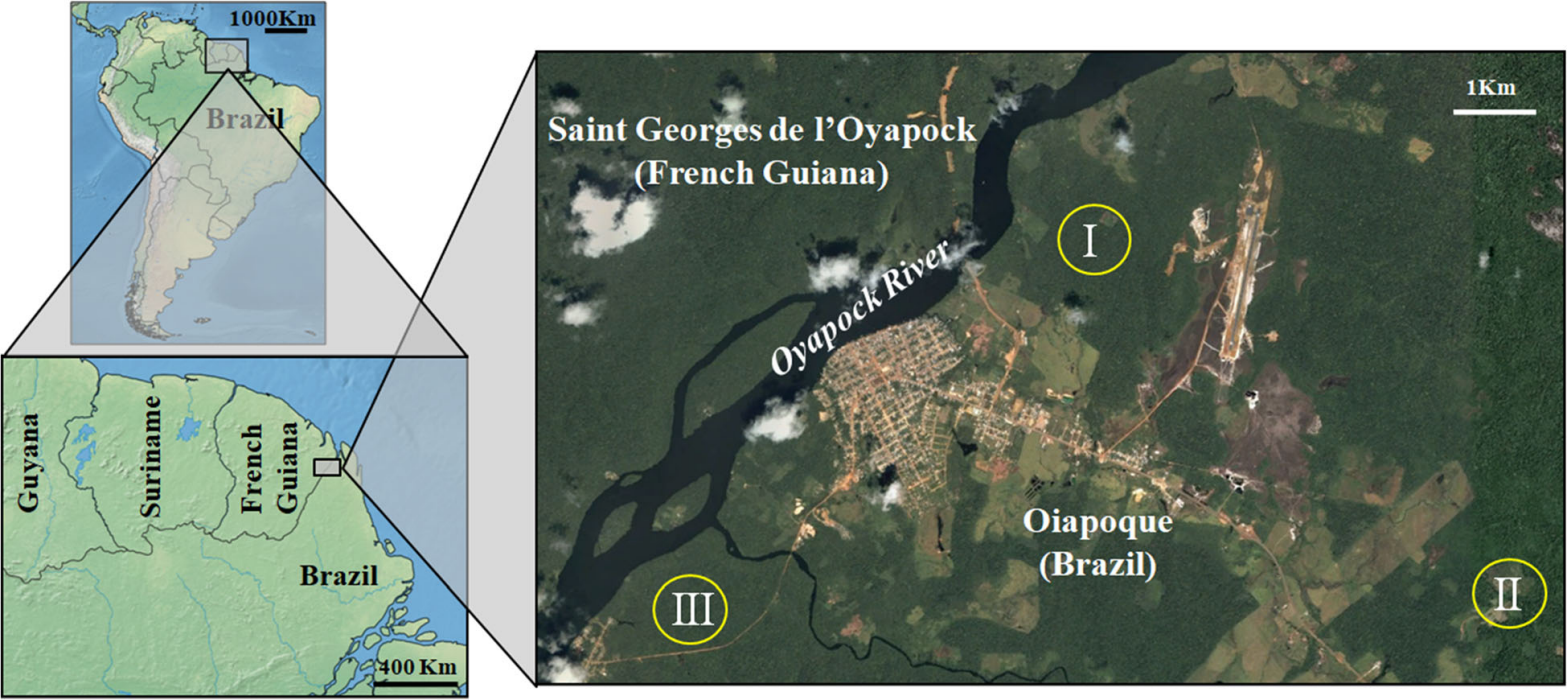
Study area on the outskirts of the Oiapoque urban area (lower Oyapock River Basin), Amapá State, Brazil, bordering French Guiana. *Abbreviations*: I, Vila Vitória Road; II, Highway BR156-Km6; III, Clevelândia do Norte Road

Located in the lower Oyapock River Basin, the urban area of Oiapoque is surrounded by different forested environments with slightly distinct ecological characteristics. Thus, three “terra-firme” (dry-land) forested sites, approximately 7 km apart, were selected for sampling as follows (Fig. 1):

(i) Vila Vitória Road (03°51'28.1"N, 51°48'41.3"W): a recently opened road that provides eastern access from Oiapoque to Vila Vitória. The sampling site shows minimal evidence of human activity and is considered well preserved.
(ii) Highway BR156-Km6 (03°49'21.0"N, 51°45'59.6"W): an impacted area in southern urban Oiapoque with evidence of human activities, such as wood extraction.
(iii) Clevelândia do Norte Road (3°49'4.14"N, 51°51'6.35"W): an old colonized area on the western side of urban Oiapoque, where the original vegetation was partially suppressed and replaced by secondary forest. It is currently an environmentally protected area by the Brazilian Army.

### Captures

Systematic field expeditions were initially performed during 2015–2016 to provide information on the predominantly rainy (February-May) and dry (September-November) Guianan/Amazonian seasons of the three sampling sites. Phlebotomines were obtained by surveying a horizontal transect from the edge inside each sampling site’s forested area, using a multi-trapping approach described elsewhere [8]. The approach comprised captures using CDC light traps (*John* W. *Hock Company*, Gainesville, USA*)* set up from 6:00 h to 18:00 h at 1.5 m (ground level; *n* = 8 traps/night) and at 20 m (canopy level; *n* = 2 traps/night), captures from 6:00 h to 20:00 h with modified Shannon black and white colored cloth [19], and manual aspiration on tree bases from 6:00 h to 20:00 h.

### Processing and identification of phlebotomines

Phlebotomines were immediately processed in the field laboratory. Females were dissected under sterile conditions [20]. Flagellate infection was semi-quantified (in a cross ‘+’ scale) according to Freitas et al. [21], and parasite development was classified by Lainson & Shaw [22]. The guts of infected females were triturated and inoculated into Difco^B45^ culture media (Becton, Dickinson and Company, Franklin Lakes, USA) to isolate the parasites. Phlebotomine species were identified under fresh conditions using morphological characteristics. Unidentified specimens were processed for mounting on glass slides using Canada balsam. Morphology and taxonomic criteria, terminology and generic abbreviations were adopted following Galati [23] and Galati et al. [24].

### Investigation of phlebotomine blood sources

Intestines dissected from engorged females were macerated in PBS (pH 7.2, 0.001 M) and stored at -20 °C until processing by *Enzyme*-Linked Immunosorbent Assay according to Afonso et al. [25]. Based on local observation and the antisera available for testing, the panel chosen comprised bird, armadillo, opossum, dog, rodent and human antisera, obtained from the Immunodiagnostics Laboratory, Department of Biological Science, Escola Nacional de Saúde Pública Sérgio Arouca, FIOCRUZ, Brazil.

### Investigation of ACL cases

Patients residing the study area who required diagnosis in the field laboratory (Oiapoque) or in the Ralph Lainson Leishmaniasis Laboratory, Instituto Evandro Chagas (Ananindeua, Brazil), were investigated according to the standard procedures of the Programa de Vigilância e Controle da Leishmaniose Tegumentar Americana (ACL Surveillance and Control Programme, Brazil). When clinically epidemiologically suggestive, patients were diagnosed by parasitological demonstration (Giemsa-stained smears of exudates from ACL lesions), the Montenegro skin test (inactivated promastigotes of *L.* (*V*.) *braziliensis* - MHOM/BR/M17323 - 1 × 10^7^ parasites/ml) and parasite isolation (inoculating exudates from ACL lesions in Difco^B45^ media) [2, 26].

### *Leishmania* spp. characterization

In both cases (phlebotomines and ACL patients), *Leishmania* DNA was obtained from successfully isolated strains. If no growth or contamination occurred, parasites were recovered from the remaining dissection slide contents (phlebotomines) or Giemsa-stained slides (ACL patients) using the DNeasy Tissue and Blood Kit (Qiagen, Hilden, Germany). Species were characterized by polymerase chain reaction-restriction fragment length polymorphism analysis (PCR-RFLP) of a 615 bp region of the *RNA polymerase II* gene amplified using the primers RPOF2 (5'-AGA ACA TGG GCG GCC-3') and RPOR2 (5'-CGA GGG TCA CGT TCT TG-3') and digested with *TpsR*I and *Hga*I endonucleases following previously established and validated methodology [27]. Digestion profiles were compared with those of the WHO *Leishmania* reference strains occurring in the Guiana Shield.

### Environmental assessment and data analysis

Forest cover degree was estimated using digital hemispherical images captured 1.6 m above ground, pointing directly upward in the CDC-ground trap sites (8 images per sampling site). Canopy coverage in each image was determined using CAN-EYE v.6.314 hemispherical image analysis software. Microclimate parameters (temperature and humidity) were measured using data loggers placed in the CDC traps. Species composition at the three sampling sites was analyzed using the Shannon-Wiener diversity index (*H*’) using PAST version 2.12 software [28]. All comparisons were performed using Student’s t*-*test to determine significance level (*P* ≤ 0.05). Sampling effort, species infection rate (SIR) and number of phlebotomines per hour were calculated according to Souza et al. [8].

## Results

### Phlebotomine composition/Environmental assessment

From the three sample areas, 9119 phlebotomines were captured during 2015–2016, belonging to 48 species. Among the 15 genera identified, *Psychodopygus* and *Psathyromyia* had the highest number of species (10 spp. and 9 spp., respectively). The composition was dominated (74.8%) by *Nyssomyia umbratilis* (29.65%), *Trichopygomyia trichopyga* (28.50%) and *Evandromyia infraspinosa* (16.36%) (Table 1). Area I had more species [40 spp.; specimens captured (*n*) = 3905; Shannon’s diversity index (*H*) *=* 2.251] than II (33 spp.; *n* = 2377; *H* = 1.857) and III (38 spp.; *n* = 2837; *H =* 1.662). The t*-*test for diversity was significant for all comparisons between sample sites (*t*_(3)_ = 11.1, *P* = 0.008).

**Table 1:** Phlebotomine species compositions at three sampling sites surveyed during four 12-day field expeditions in Oiapoque, Amapá, Brazil (2015–2016). Species found infected by flagellates are shown in bold, with the number of specimens found infected with flagellates in parentheses

No.	Species	I		II		III		Total		SIR
		**♀♀**	**♂♂**	**♀♀**	**♂♂**	**♀♀**	**♂♂**	*N*	%	
1	***Nyssomyia umbratilis*** (28)	482 (19)	426	725 (8)	479	355 (1)	237	2704	29.65	1.79
2	*Trichopygomyia trichopyga*	456	522	128	124	655	714	2599	28.50	–
3	***Evandromyia infraspinosa*** (3)	344 (2)	352	233 (1)	122	255	186	1492	16.36	0.36
4	*Trichophoromyia ininii*	172	158	27	34	13	24	428	4.69	–
5	*Psathyromyia aragaoi*	108	121	16	9	39	47	340	3.73	–
6	*Psyshodopygus maripaensis*	68	11	130	9	2	–	220	2.41	–
7	***Sciopemyia sordellii*** (1)	24 (1)	14	18	19	45	15	135	1.48	1.14
8	*Micropygomyia rorotaensis*	67	36	6	3	19	2	133	1.46	–
9	*Psyshodopygus ayrozai*	59	25	29	15	4	–	132	1.45	–
10	*Bichromomyia flaviscutellata*	69	30	1	3	7	1	111	1.22	–
11	*Nyssomyia anduzei*	4	1	4	–	64	18	91	1.00	–
12	*Evandromyia williamsi*	17	5	7	36	5	1	71	0.78	–
13	*Evandromyia brachyphalla*	41	14	5	–	4	5	69	0.76	–
14	***Viannamyia furcata*** (2)	17 (1)	10	18 (1)	11	11	1	68	0.75	4.34
15	*Viannamyia tuberculata*	19	3	26	3	5	–	56	0.61	–
16/17	*Pressatia choti/ Pr. trispinosa*	12	8/21	8	–/1	2	–/1	53	0.58	–
18	*Evandromyia* sp. of Baduel	11	19	8	10	–	–	48	0.53	–
19	*Nyssomyia pajoti*	9	2	3	–	15	8	37	0.41	–
20	***Sciopemyia fluviatilis*** (2)	4 (2)	16	4	3	4	–	31	0.34	16.6
21	*Psathyromyia inflata*	8	13	4	6	–	–	31	0.34	–
22	*Lutzomyia spathotrichia*	2	1	17	11	–	–	31	0.34	–
23	*Evandromyia monstruosa*	13	3	10	–	2	2	30	0.33	–
24	***Migonemyia migonei*** (2)	14 (2)	8	1	–	3	1	27	0.30	11.1
25	*Psyshodopygus hirsutus*	6	2	8	6	2	1	25	0.27	–
26	***Pintomyia damascenoi*** (1)	4 (1)	–	7	6	2	–	19	0.21	–
27	*Psyshodopygus davisi*	4	4	1	2	4	–	15	0.16	–
28	***Psathyromyia dendrophyla*** (1)	3 (1)	4	3	1	4	–	15	0.16	10
29	*Psychodopygus claustrei*	4	7	–	–	2	1	14	0.15	–
30	*Pschodopygus corrosoniensis*	3	1	–	–	8	2	14	0.15	–
31	*Psathyromyia bigeniculata*	4	1	5	1	1	1	13	0.14	–
32	*Psathyromyia dreisbachi*	–	–	–	–	9	4	13	0.14	–
33	*Trichophoromyia ubiquitalis*	–	–	–	–	5	8	13	0.14	–
34/35	*Brumptomuyia travassosi/Br. pentacantha*	4	1/1	1	–	2	2/–	7	0.07	–
36	*Psyschodopygus amazonensis*	–	–	2	2	1	–	5	0.05	–
37	*Psychodopygus paraensis*	3	–	–	–	–	1	4	0.04	–
38	*Psathyromyia punctigeniculata*	–	–	–	3	1	–	4	0.04	–
39	*Trichopygomyia dasypodogeton*	3	–	–	–	–	–	3	0.03	–
40	*Pintomyia pacae*	1	–	–	–	1	–	2	0.02	–
41	*Pintomyia serrana*	1	–	–	–	–	1	2	0.02	–
42	*Psathyromyia lutziana*	2	–	–	–	–	–	2	0.02	–
43	*Psathyromyia abonnenci*	1	1	–	–	–	–	2	0.02	–
44	*Psathyromyia barrettoi barrettoi*	–	–	1	1	–	–	2	0.02	–
45	*Evandromyia begonae*	–	–	–	–	–	1	1	0.01	–
46	*Micropygomyia* (Pilosa Series)	1	–	–	–	–	–	1	0.01	–
47	*Psychodopygus bispinosus*	–	–	–	–	1	–	1	0.01	–
48	*Psychodopygus carrerai*	–	–	1	–	–	–	1	0.01	–
	Total	2064	1841	1457	920	1552	1,285	9119	100.00	–
		3905		2377		2837				0.78
	Taxa (*S*)	40		33		38		–	–	–
	Shannon’s *H*^a^	2.251		1.857		1.662		–	–	–

*Abbreviations*: *I* Vila Vitória Road, *II* Highway BR156-Km6, *III* Clevelândia do Norte Road, ♀♀ females, ♂♂ males, *SIR* species infection rate (flagellates), *n/n* number of males, while indistinguishable females, *N* total number

^a^The t*-*test for diversity was significant for all comparisons between sample sites (*t*_(3)_ = 11.1, *P* = 0.008)

**Fig. 2:**
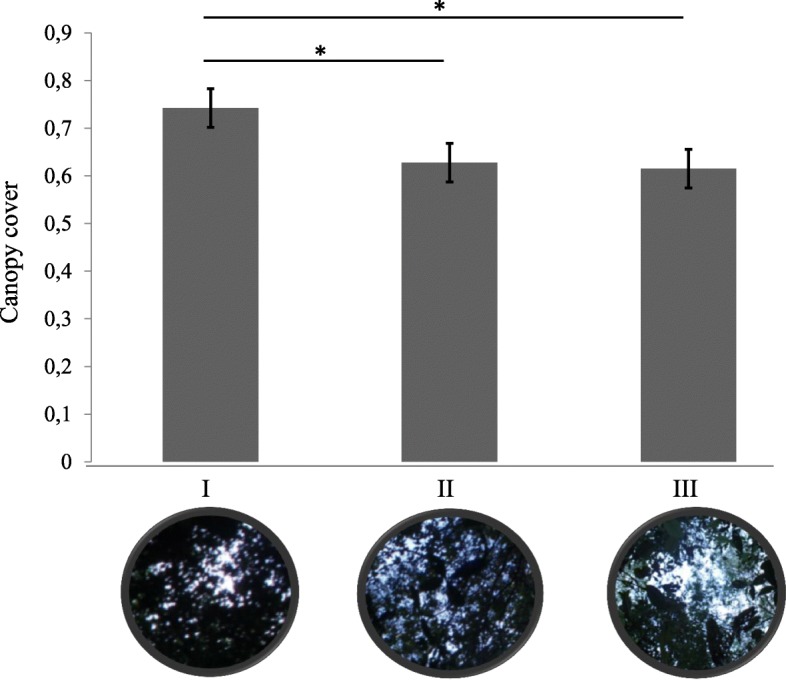
Estimation of canopy cover degree at the three surveyed ecotopes (forested areas) on the outskirts of the Oiapoque urban area (lower Oyapock River Basin), Amapá State, Brazil, bordering French Guiana. Asterisk indicates significant differences (*P* ≤ 0.05). *Abbreviations*: I, Vila Vitória Road; II, Highway BR156-Km6; III, Clevelândia do Norte Road

Estimation of canopy cover differed significantly between areas I and II (*t*_(2)_ = 11.83, *P* = 0.004) and between I and III (*t*_(2)_ = 21.46, *P* = 0.002) (Fig. 2). Average temperature (°C) and humidity (%) registered in the tree sampled sites were, respectively: I (27.4 °C; 82.9%); II (26.6 °C; 77.1%); III (25.0 °C; 85.7%). However, no statistically significant differences were found between average temperature (*t*_(3)_ = 1.77, *P* = 0.63) or humidity (*t*_(3)_ = 1.95, *P =* 0.21).

Based on 4608 h of CDC ground, 1152 h of CDC canopy trap, 48 h of white Shannon, 48 h of black Shannon, and 24 h of aspiration on tree bases (total sampling effort: 5880 h), white Shannon traps rendered 5.6 phlebotomines per hour. The greatest number of phlebotomines found per hour for these capture methods included *Ny. umbratilis* in the CDC canopy trap (0.96 ♀♀), white Shannon trap (0.93 ♀♀), and on aspiration on tree bases (1.16 ♀♀; 1.62 ♂♂). *Evandromyia infraspinosa* was also found on the black Shannon trap (0.97 ♀♀), and *Ps. s. maripaensis* was found on the white Shannon trap (2.29 ♀♀). These three species attempted to bite the professionals during capture (Table 2).

**Table 2:** Averages for the ten most frequently captured species per hour compiled for the three sampling sites surveyed during four 12-day field expeditions in Oiapoque, Amapá, Brazil (2015–2016). Highest individual values found (above 0.9) are shown in bold

No.	Species	Capture method^a^										
		CDC ground		CDC canopy		White Shannon		Black Shannon		Tree bases		Total^b^
		♀	♂	♀	♂	♀	♂	♀	♂	♀	♂	
1	*Nyssomyia umbratilis*	0.08	0.05	**0.96** ^c^	0.74	**0.93** ^c^	0.1	0.04	–	**1.16**	**1.62**	0.46
2	*Trichopygomyia trichopyga*	0.23	0.25	0.11	0.15	0.08	0.08	0.12	0.06	–	–	0.44
3	*Evandromyia infraspinosa*	0.13	0.10	0.13	0.11	0.5^c^	0.29	**0.97** ^c^	0.54	0.04	0.08	0.25
4	*Trichophoromyia ininii*	0.04	0.04	0.00	0.01	0.04	–	0.08	–	–	–	0.07
5	*Psathyromyia aragaoi*	0.02	0.02	0.04	0.02	–	–	0.08	–	–	–	0.05
6	*Psychodopygus squamiventris maripaensis*	0.00	0.00	0.02	0.00	**2.29** ^c^	0.1	0.68^c^	0.12	0.04	–	0.03
7	*Sciopemyia sordellii*	0.02	0.00	0.02	0.01	0.04	0.04	0.12	0.04	0.08	–	0.03
8	*Psychodopygus ayrozai*	0.00	0.00	0.04	0.02	0.22^c^	–	0.22^c^	0.02	–	–	0.02
9	*Micropygomyia rorotaensis*	0.01	0.00	0.02	0.01	0.1	–	0.06	–	0.16	0.16	0.02
10	*Bicrhomomyia flaviscutellata*	0.01	0.00	0.00	0.00	0.14	0.08	0.1	–	–	–	0.01
	Other species (11–48)	0.05	0.03	0.15	0.09	0.35	0.1	0.22	0.06	0.12	0.29	0.12
	Total	0.62	0.54	1.54	1.21	4.7	0.81	2.75	0.05	1.62	2.16	1.55
	Total (♀ + ♂)	1.17		2.76		5.6		3.6		3.79		–

^a^Based on 4608 h CDC ground; 1152 h CDC canopy; 48 h white Shannon; 48 h black Shannon; and 24 h tree bases

^b^Total sampling effort: 5880 h

^c^Specimens found attempting to bite

### Parasite infections and *Leishmania* typing

Forty-eight parasite infections were found, representing 40 flagellates, five nematodes (Nemathelminthes) and three gregarines (Apicomplexa). Flagellate infections were detected in *Nyssomyia umbratilis* (*n* = 28), *Evandromyia infraspinosa* (*n* = 3), *Migonemyia migonei* (*n* = 2), *Sciopemyia fluviatillis* (*n* = 2) *Viannamyia furcata* (*n* = 2), *Psathyromyia dendrophyla* (*n* = 1), *Sciopemyia sordellii* (*n* = 1) and *Pintomyia damascenoi* (*n* = 1). Nematode infections occurred in *Evandromyia williamsi* (*n* = 2), *Psathyromyia aragaoi* (*n* = 1), *Evandromyia monstruosa* (*n* = 1) and *Ps. s. maripaensis* (*n* = 1). Gregarines were found in *Bi. flaviscutellata* (3).

Twelve flagellate strains were successfully isolated (Table 3), all without visible blood meals, and with infections varying from ++ to ++++. Isolates occurred in 10 *Ny. umbratilis* at sites I (*n* = 3), II (*n* = 6) and II (*n* = 1) and in one *Sc. fluviatilis* (site I) and one *Vi. furcata* (site I).

**Table 3:** Strains of *Leishmania* and other flagellates isolated *in vitro* from naturally infected phlebotomine specimens captured at the three sampled sites in Oiapoque, Amapá, Brazil (2015–2016)

No.	Species	IEC code	Capture site	Capture method	Infection^a^	PCR-RFLP result - WHO code
1	*Nyssomyia umbratilis*	M31681	II	CDC ground	+++	*L*. (*V.*) *guyanensis* - IUMB/BR/2015/M31681
2	*Nyssomyia umbratilis*	M32146	I	CDC ground	+++	*L*. (*V.*) *guyanensis* - IUMB/BR/2016/M32146
3	*Nyssomyia umbratilis*	M32149	I	Tree bases	++	*L*. (*V.*) *guyanensis* - IUMB/BR/2016/M32149
4	*Nyssomyia umbratilis*	M32152	I	CDC canopy	++++	*L*. (*V.*) *guyanensis* - IUMB/BR/2016/M32152
5	*Nyssomyia umbratilis*	M32154	II	CDC ground	+++	*L*. (*V.*) *guyanensis* - IUMB/BR/2016/M32154
6	*Nyssomyia umbratilis*	M32156	II	CDC canopy	++++	*L*. (*V.*) *guyanensis* - IUMB/BR/2016/M32156
7	*Nyssomyia umbratilis*	M32157	II	CDC canopy	++++	*L*. (*V.*) *guyanensis* - IUMB/BR/2016/M32157
8	*Nyssomyia umbratilis*	M32158	II	CDC canopy	++	*L*. (*V.*) *guyanensis* - IUMB/BR/2016/M32158
9	*Nyssomyia umbratilis*	M32159	II	CDC canopy	++	*L*. (*V.*) *guyanensis* - IUMB/BR/2016/M32159
10	*Nyssomyia umbratilis*	M32160	III	CDC ground	+++	*L*. (*V.*) *guyanensis* - IUMB/BR/2016/M32160
11	*Sciopemyia fluviatilis*	M32316	I	CDC ground	++	Unconclusive - IFLU/BR/2016/M32316
12	*Viannamyia furcata*	M32652	I	CDC ground	+++	Unconclusive - IFUR/BR/2016/M32652

*Abbreviations*: *I* Vila Vitória Road, *II* Highway BR156-Km6, *III* Clevelândia do Norte Road

^a^Parasites per field (×40 objective): ++, 6–20; +++, 21–40; ++++, > 40

**Fig. 3:**
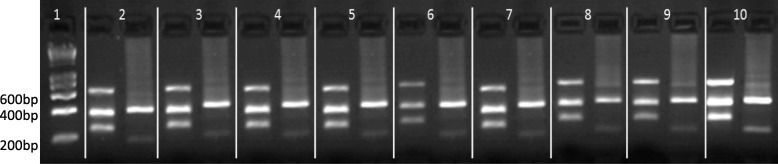
PCR-RFLP analysis (primers RPOF2/RPOR2, restriction enzymes *TspR*I/ *Hga*I) of *Leishmania* (*Viannia*) *guyanensis* isolates from phlebotomine species and an ACL case from the outskirts of the Oiapoque urban area (lower Oyapock River Basin), Amapá State, Brazil, bordering French Guiana, compared with that of the WHO reference strain. The two columns for each sample represent the digestion products of *TspR*I and *Hga*I, respectively. Lane 1: molecular weight marker Smart Ladder; Lane 2: IUMB/BR/2015/M31681; Lane 3: IUMB/BR/2016/M32146; Lane 4: IUMB/BR/2016/M32149; Lane 5: IUMB/BR/2016/M32156; Lane 6: IUMB/BR/2016/M32158; Lane 7: IUMB/BR/2016/M321597; Lane 8: IUMB/BR/2016/M32160; Lane 9: MHOM/BR/2017/M32218; Lane 10: *L.* (*V.*) *guyanensis* WHO reference strain (MHOM/BR/1975/M4147)

All isolates from *Ny. umbratilis* exhibited a PCR-RFLP profile identical to that of the *L.* (*V.*) *guyanensis* WHO reference strain (MHOM/BR/1975/M4147) (Fig. 3), while those from *Sc. fluviatilis* and *Vi. furcata* were inconclusive.

**Fig. 4:**
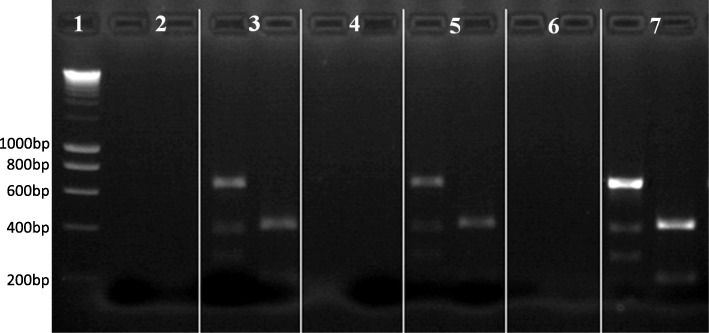
PCR-RFLP analysis (primers RPOF2/RPOR2, restriction enzymes *TspR*I/ *Hga*I) of a 615 bp amplified fragment of the *RNA polymerase II* gene from *Leishmania* (*Viannia*) *guyanensis* DNA from material remaining on glass dissection slides of infected phlebotomine specimens captured in Oiapoque, Amapá Brazil, compared with that of the WHO reference strain. The two columns for each sample represent the digestion products of *TspR*I and *Hga*I, respectively. Lane 1: molecular weight marker Smart Ladder; 1 kb; Lane 2: negative sample; Lane 3: *Ny. umbratilis*-M32153; Lane 4: negative sample; Lane 5: *Ev. infraspinosa-*M32155; Lane 6: negative control; Lane 7: WHO reference strain (MHOM/BR/1975/M4147)

Twenty-eight infections were not isolated. The PCR-RFLP for the remaining DNA fixed on the glass dissection slides allowed characterizing *L.* (*V.*) *guyanensis* from one *Ny. umbratilis* and two *Ev. infraspinosa* specimens (Fig 4).

### Blood sources

One hundred thirty-eight guts were tested, and 20 (14.4%) reacted to at least one antiserum on the available panel (Table 4). Positive species comprised *Ny. umbratilis* (*n* = 12), *Ps. s. maripaensis* (*n* = 4), *Ps. claustrei* (*n* = 2), *Bichromomyia flaviscutellata* (*n* = 1) and *Ev. infraspinosa* (*n* = 1). The sampling site with the most engorged flies was I (*n* = 16). The engorged specimens were mostly found in tree bases (*n* = 7), followed by the CDC ground trap (*n* = 4) and lastly the Shannon trap (*n* = 1).

**Table 4:** Phlebotomine specimens captured at three sampling sites in Oiapoque, Amapá, Brazil (2015–2016), tested by ELISA for blood sources and found positive for at least one antiserum from the available panel

No.	Species	Site	Method	Blood source^a^
1	*Nyssomyia umbratilis*	III	CDC canopy	Bird
2	*Nyssomyia umbratilis*	III	CDC canopy	Bird
3	*Psychodopygus claustrei*	I	CDC ground	Dog, opossum, rodent
4	*Psychodopygus claustrei*	I	CDC ground	Armadillo
5	*Bichromomyia flaviscutellata*	I	CDC canopy	Bird
6	*Psychodopygus squamiventris maripaensis*	I	CDC canopy	Armadillo
7	*Psychodopygus squamiventris maripaensis*	II	CDC canopy	Armadillo
8	*Psychodopygus squamiventris maripaensis*	II	CDC canopy	Armadillo
9	*Evandromyia infraspinosa*	I	CDC ground	Armadillo
10	*Nyssomyia umbratilis*	I	Tree bases	Bird
11	*Nyssomyia umbratilis*	I	Tree bases	Bird
12	*Nyssomyia umbratilis*	I	Tree bases	Bird
13	*Nyssomyia umbratilis*	I	Tree bases	Dog, opossum
14	*Psychodopygus squamiventris maripaensis*	I	Shannon	Armadillo
15	*Nyssomyia umbratilis*	I	Tree bases	Bird
16	*Nyssomyia umbratilis*	I	Tree bases	Bird, dog, armadillo
17	*Nyssomyia umbratilis*	I	CDC canopy	Bird
18	*Nyssomyia umbratilis*	I	CDC ground	Man
19	*Nyssomyia umbratilis*	I	Tree bases	Armadillo
20	*Nyssomyia umbratilis* ^b^	I	CDC canopy	Bird

*Abbreviations*: *I* Vila: Vitória Road, *II* Highway BR156-Km6, *III* Clevelândia do Norte Road

^a^Antisera panel: dog, bird, opossum, man, armadillo and rodent

^b^Positive sample with flagellates morphologically compatible with *Leishmania* sp.

Species with the greatest numbers of identified blood sources included *Ny. umbratilis* (bird, dog, armadillo, opossum and human), followed by *Ps. claustrei* (dog, opossum, rodent and armadillo)*.* The other positive species had only one blood source: *Bichromomyia flaviscutellata* (bird); *Ps. s. maripaensis* (armadillo); and *Ev. infraspinosa* (armadillo).

Species with more than one blood source in the same specimen included *Ny. umbratilis* (sample 13: dog and opossum; sample 16: bird, dog and armadillo) and *Ps. claustrei* (sample 3: dog, opossum and rodent). One *Ny. umbratilis* naturally infected with flagellates morphologically compatible with *Leishmania* spp. was positive for bird antiserum.

### American cutaneous leishmaniasis cases

During 2015–2016, ten patients belonging to the Guiana Shield asked for ACL diagnostics in our laboratory, and eight believed that they had been infected on the Brazilian-French Guianan/Oyapock border (six from gold mining and two from agricultural settlements) (Table 5). All *Leishmania* strains were isolated and characterized as *L.* (*V*.) *guyanensis*. A Brazilian agricultural settlement with *L.* (*V.*) *guyanensis* isolated from an ACL patient (MHOM/BR/2017/M32218) is located in site I, near the forested area subjected to the captures.

**Table 5:** *Leishmania* (*Viannia*) *guyanensis* strains isolated from cutaneous lesions of patients treated at the Ralph Lainson Leishmaniasis Laboratory (IEC/SVS/MS) (2015–2017) who declared the Brazil-French Guiana border as the probable place of infection

Mnemonic	Infection site	No. of lesions (location)	IDRM (mm)	WHO code
FCF	Vila Vitória (BR)	2 (face/neck)	12 × 12	MHOM/BR/2017/M32218
HMLR	Gold mining (FG)	1 (foot)	12 × 12	MHOM/BR/2016/M31987
MRP	Gold mining (FG)	2 (hand/leg)	7 × 7	MHOM/BR/2016/M32048
ARSN	Gold mining (FG)	1 (arm)	8 × 8	MHOM/BR/2015/M31041
PSLS	Gold mining (FG)	Disseminated	10 × 10	MHOM/BR/2015/M31157
HS	Gold mining (FG)	1 (thorax)	12 × 12	MHOM/BR/2015/M31498
OSM	Régina (FG)	1 (leg)	10 × 10	MHOM/BR/2015/M32273
LMSJr	Gold mining (FG)	6 (leg (4), arm (1), neck (1))	17 × 17	MHOM/BR/2015/M32382

*Abbreviations*: *BR* Brazil, *FG* French Guiana

Figure 3 shows a PCR-RFLP analysis of *Leishmania* isolates from infected phlebotomines and a human ACL case compared against the WHO *L.* (*V*.) *guyanensis* reference strain.

## Discussion

Few studies have been conducted on ACL ecology in the lower Oyapock basin. The first available information on that region (the French Guianan side) came from entomological studies conducted in the 1940s and 1950s, which provided much information on phlebotomine taxonomy and ecology [29]. The available commented checklist including that region can be found elsewhere [30] as a metabarcoding-based local inventory [31]. Evidence on ACL etiology was only provided recently [15].

With reasonable sampling efforts (5880 h) for the multi-trapping approach, the present findings showed high species diversity (48 spp.). We preferred to use different capture methods because, although light-baited suction traps are one of the most widely used tools for vector surveillance, they have biases and limitations in terms of their effect on collection efficiency, population data, and pathogen detection [32]. Multi-trapping approaches with large samplings may offer a broader picture on the surveyed fauna, as shown by Souza et al. [33] in the Lower Amazon Basin (68 spp.) and by Freitas et al. [21] (46 spp.) and Souza et al. [8] (63 spp.) in Amapá. However, the use of CDC light traps in long term surveys and/or with strategical placement (i.e. with spatial stratification biasing to find feeding/resting sites) may, in part, supply some of these limitations. The present results are also reasonably compatible with ‘CDC trap-based’ surveys recently conducted in the Guiana Shield by Rotureau et al. [34] (46 spp.) and Fouque et al. [35] (38 spp.) in French Guiana, as well as by Furtado et al. [36] (45 spp.) in Amapá. Compiled information shows that approximately 84 species are registered in Amapá, and *Brumptomyia pentacantha* was a newly recorded species for that state. In Brazil, this species was recorded only in Pará, Acre, Rondônia and Mato Grosso states [8, 36–45].

Despite high overall species diversity, numerical domination (74.8%) of only three species was expected. Studies on forested environments have shown a phlebotomine fauna generally composed of a few dominant species and many species with few specimens [46, 47].

Differences in the degree of canopy cover between the three sampling sites were congruent with their respective Shannon indices (*H*), suggesting forest cover as an eligible variable for maintaining species diversity, although deforestation associated with human settlements can also produce environmental conditions suitable for maintaining the life-cycles of several sand fly species that are adaptable to these environments [48, 49]. Conversely, high SIR was found at sites with high degrees of canopy cover. Dense and humid substrate provided by a well-covered canopy may contribute to vector/host availability. In addition, low light penetration in the denser forest can provide suitable conditions for vector/host movements, as observed with the inverse correlation of phlebotomine density on light traps *versus* moonlight [50]. However, a minimum light is important for these insects to fly [51]; thus, vector-host interactions may result from equilibrium between these factors.

Numerous nematodes were found in the body cavity of five phlebotomine species. Although these flies were captured at different sampling sites and different vertical strata (ground/canopy level), some entomopathogenic nematode species infect phlebotomines on the ground, during the larval stage [52], suggesting that these infected flies may share the same breeding site.

Gregarines found in *Bi. flaviscutellata* (three specimens) were morphologically similar to *Psychodiella* sp. oocysts, although only molecular sequencing could confirm the species. Insect-host specificity between *Bi. flaviscutellata* and the gregarine species supports the hypothesis of a long, strong coevolutionary association between them [53].

Only 14.4% of blood-fed phlebotomines tested were positive by ELISA. This result could be attributed to the low blood content in the specimens as well as the blood recuperation procedure for the dissected slides, which may have contributed to the loss of material. Another possibility is the presence of animal blood; this was not accounted for by the available test panel since anteaters and sloths, for example, are presumably present in the Guianan ecosystem, acting as potential reservoir hosts of *L.* (*V*.) *guyanensis* [54, 55]. These results provided a better understanding of the biology of five phlebotomine species.

Undoubtedly, *Ny. umbratilis* is closely associated with *L.* (*V*.) *guyanensis* and has been consequently implicated as the main ACL vector in Oyapock based on data consistent with its well-recognized regional importance in the Guiana Shield [14] and in the wide Amazonian region [7]. Infection rates for this fly vary greatly in the literature, with some being consistent with the present findings [8, 56, 57]. Higher rates are usually biased by captures performed in the dry season [58] or supporting the dissection of fed and gravid females [21]. Infected specimens were captured in both levels of CDC traps and tree bases; however, they may have been infected at other sites. A natural vertical migration of these flies is well documented [13] and may explain dissociation movements between infection/resting sites as supposed for *L.* (*V.*) *naiffi* in the Lower Amazonian basin, where a canopy of *Ps. davisi* was found infected by that parasite, whose only recognized potential reservoir host is the terrestrial armadillo, *Dasypus novemcintus* [32].

Blood contents from *Ny. umbratilis* reacted mainly with bird antisera (9/12). The role of birds in the population dynamics of phlebotomine species has been discussed [59–62]. *Nyssomyia umbratilis* being found with bird blood and concomitantly with a flagellate (likely leishmanine parasites) infection could be an occasional finding or suggests that this blood source provided suitable conditions for *L.* (*V*.) *guyanensis* development, as has been demonstrated in experiments between *Gallus gallus* blood and *L.* (*L.*) *mexicana* [63]. The findings on the eclectic food habits of *Ny. umbratilis* are consistent with those of other studies [64, 65]. Rodents, for example, appear to be alternative blood sources in disturbed environments [66], contributing to a possible groundward vertical migration of this traditionally canopy-loving phlebotomine species, as presumed to occur in a hydroelectric system-affected area of Jari River Basin [36].

Three infections found in *Ev. infraspinosa* were not isolated, but two were successfully characterized as *L.* (*V*.) *guyanensis* from the DNA content on the dissection slides. Considerable infections (++) and absence of blood observed with these peripylaric parasites suggest the necessity to continuously investigate this fly’s possible involvement in the *L.* (*V*.) *guyanensis* enzootics. In addition, this species was frequent in Shannon captures (mainly in the black cloth, 0.97 females/h), with some specimens attempting to bite the professionals. It was impossible to determine whether *Ev. infraspinosa* could feed on potential *L.* (*V*.) *guyanensis* reservoirs. The present results indicate that this phlebotomine can feed on armadillos. The rodent *Dasyprocta leporina* is the only known blood source for this species [65] although anuran trypanosomatid isolated from this species from the western Brazilian Amazon suggests it feeds on cold-blooded rather than warm-blooded vertebrates [67].

In addition to *Ny. umbratilis* and *Ev. infraspinosa*, flagellate infections have been found in *Mi. migonei*, *Sc. fluviatilis*, *Sc. sordellii* and *Vi. furcata*. Negative PCR-RFLP for the other infected specimens suggested low DNA for *Leishmania-*typing or that they were other trypanosomatids. The apparent high infection rates of some of these species may have been biased by the low number of dissected females and thus cannot yield conclusive findings.

Other phlebotomine species were found with flagellates. The trypanosomatid isolated from *Sc. fluviatilis* will be further characterized. Parasites found in the blood of *Pa. dendrophyla* should be cautiously interpreted; this species shares the same ecotope as *Ny. umbratilis*, and some specimens likely receive occasional parasite ingestions, as observed by Freitas et al. [21]. These considerations can be extended to *Vi. furcata* and *Pi. damascenoi* [13].

Although no *Leishmania* infections were found for the following two fly species, they should still be discussed as putative vectors in the lower Oyapock River Basin based on the current Guianan/Amazonian ACL epidemiological background:

(i) *Psychodopygus s. maripaensis* has been included on the long list of possible vectors of *L.* (*V*.) *naiffi* based on infection findings in Régina, French Guiana [35] and Serra do Navio (AP) [8], extending its epidemiological relevance in other Brazilian/Guianan regions [36]. In addition, the *Ps. s. maripaensis* specimens tested for blood sources reacted positively to antiserum from an armadillo, the recognized potential reservoir of *L.* (*V.*) *naiffi.* Interestingly, DNA from *L.* (*V*.) *braziliensis* was detected in a pooled sample of *Ps. s. maripaensis* [referred to as *P. squamiventris* (*s.l.*)] in Sabajo Heuvels, Suriname, suggesting an additional putative vector role [68]. However, *L*. (*V*.) *braziliensis* transmission in Oyapock remains unclear. The most females found per hour in our Shannon captures (2.29; white colored cloth), with some attempting to bite the professionals, demonstrates the aggressive behavior of this species.

(ii) *Bichromomyia flaviscutellata* is the vector of *L.* (*L*.) *amazonensis* [7]*.* The presence of this sand fly in the surveyed sites, mainly site I, is noteworthy because of the pathological spectrum of its associated parasite [9] despite only the cutaneous form being documented in French Guiana [14, 15]. The synanthropic behavior of the Guianan population of *Bi. flaviscutellata*, which appears to adapt to environments under ecological pressures and human-made modifications [36, 69], has been documented. One *Bi. flaviscutellata* from a CDC canopy trap was positive for bird antiserum, raising the hypothesis that this species could migrate vertically to search for alternative blood sources. Domestic birds, such as chickens, may be attractive for this phlebotomine species, triggering it to adapt to modified environments. Our preliminary results from a peridomiciliary-forest stratification study have shown that some *Bi. flaviscutellata* specimens are captured outdoors, where animal shelters can stimulate phlebotomines to cross a 200 m gradient between the forest border and households (Vasconcelos dos Santos, unpublished data).

Most ACL isolates (6/8) were from patients infected while gold mining, showing that *L.* (*V*.) *guyanensis* ACL hotspots may be concentrated in these environments (upper Oyapock River Basin) [14, 15, 18]. Conversely, the present entomological results showed considerable infection rates of enzootics near urban cities, in which less economically attractive periurban forests (absence of gold mining) may reflect less human exposure to the disease (and consequently few ACL cases) in that area.

## Conclusions

Our findings show that ACL transmission in the Oyapock River Basin reflects the Guianan/Amazonian classical ecosystem, where *Ny. umbratilis* remains the main vector. A putative alternative transmission by *Ev. infraspinosa* is possible, but circumstantial parasite ingestion is also likely, as seen with other biologically compatible phlebotomine species cohabiting the same potential *L.* (*V.*) *guyanensis* reservoir ecotopes. Conversely, epidemiological relevance of these putative alternative transmission cycles cannot be estimated with certainty. Literature-based evidence indicates that others fly species, such as *Ps. s. maripaensis* and *Bi. flaviscutellata* are also epidemiologically relevant, and we included them on the priority list for vector surveillance in the lower Oyapock basin. Local studies on ACL enzootics should be encouraged, since each an ecological mosaic is unique. The ACL etiology shows that the transmission pattern in the upper Oyapock may differ slightly from the lower basin, but only further surveys of the former environment can confirm this hypothesis.

## Abbreviations

ACL: American cutaneous leishmaniasis
WHO: World Health Organization
CDC: Center of Diseases Control

## References

[1] Lainson R, Shaw JJ, Collier L, Balows A, Sussman M (2005). Leishmaniasis in the New World. Topley & Wilson’s Microbiology and Microbial Infections, 10th ed., v. 5. Parasitology.

[2] WHO (2010). Control of the leishmaniases, report of a meeting of the WHO Expert Committee on the control of leishmaniases, Geneva, 22–26 March 2010. WHO Technical Report Series, n° 949.

[3] Ready PD (2013). Biology of phlebotomine sand flies as vectors of disease agents. Annu Rev Entomol..

[4] Brazil RP, Rodrigues AAF, Andrade Filho JD (2015). Sand fly vectors of *Leishmania* in the Americas - a mini review. Entomol Ornithol Herpetol..

[5] Espinosa OA, Serrano MG, Camargo EP, Teixeira MMG, Shaw JJ (2016). An appraisal of the taxonomy and nomenclature of trypanosomatids presently classified as *Leishmania* and *Endotrypanum*. Parasitology.

[6] Killick-Kendrick R (1985). Some epidemiological consequences of the evolutionary fit between leishmaniae and their phlebotomine vectors. Bull Soc Path Exot Fil..

[7] Rangel EF, Lainson R (2009). Proven and putative vectors of American cutaneous leishmaniasis in Brazil: aspects of their biology and vectorial competence. Mem Inst Oswaldo Cruz..

[8] Souza AAA, Barata IR, Silva MGS, Lima JAN, Jennings YLL, Ishikawa EAY (2017). Natural *Leishmania* (*Viannia*) infections of phlebotomines (Diptera: Psychodidae) indicate classical and alternative transmission cycles of American cutaneous leishmaniasis in the Guiana Shield, Brazil. Parasite.

[9] Silveira FT, Lainson R, Corbett CEP (2004). Clinical and immunopathological spectrum of American cutaneous leishmaniasis with special reference to the disease in Amazonian Brazil: a review. Mem Inst Oswaldo Cruz..

[10] Lainson R, Ward RD, Shaw JJ (1976). Cutaneous leishmaniasis in North Brazil: *Lutzomyia anduzei* as a major vector. Trans R Soc Trop Med Hyg..

[11] Ward RD, Fraiha H (1977). *Lutzomyia umbratilis*, a new species of sand fly from Brazil (Diptera: Psychodidae). J Med Entomol..

[12] Lainson R, Shaw JJ, Ready PD, Miles MA, Póvoa MM (1981). Leishmaniasis in Brazil: XVI. Isolation and identification of *Leishmania* species from sand flies, wild mammals and man in north Para State, with particular reference to *L. braziliensis guyanensis* causative agent of “pian-bois”. Trans R Soc Trop Med Hyg..

[13] Ready PD, Lainson R, Shaw JJ, Ward RD (1986). The ecology of *Lutzomyia umbratilis* Ward & Fraiha (Diptera: Psychodidae), the major vector to man of *Leishmania braziliensis guyanensis* in north-eastern Amazonian Brazil. Bull Entomol Res..

[14] Rotureau B (2006). Ecology of the *Leishmania* species in the Guianan Ecoregion Complex. Am J Trop Med Hyg..

[15] Simon S, Nacher M, Carme B, Basurko C, Roger A, Adenis A (2017). Cutaneous leishmaniasis in French Guiana: revising epidemiology with PCR-RFLP. Trop Med Health..

[16] Rotureau B, Joubert M, Clyti E, Djossou F, Carme B (2006). Leishmaniasis among gold miners, French Guiana. Emerg Infect Dis.

[17] Instituto Brasileiro de Geografia e Estatística (2013). Coordenação de População e Indicadores Sociais (1st July 2013). Estimates of resident population in the Brazilian municipalities.

[18] Vasconcelos dos Santos T, MCG C, Prévot G, Silveira FT, Póvoa MM, Rangel EF. Binational burden of American cutaneous leishmaniasis in Oiapoque, Amapá State, Brazil, bordering French Guiana. Rev Soc Bras Med Trop. 2018 (In Press). 10.1590/0037-8682-0256-2018 30942256

[19] Brilhante AF, de Ávila MM, de Souza JF, Medeiros-Sousa AR, Sábio PB, de Paula MB (2017). Attractiveness of black and white modified Shannon traps to phlebotomine sand flies (Diptera, Psychodidae) in the Brazilian Amazon Basin, an area of intense transmission of American cutaneous leishmaniasis. Parasite.

[20] Ryan L, Lainson R, Shaw JJ (1987). Leishmaniasis in Brazil. XXIV. Natural flagellate infections of sand flies (Diptera: Psychodidae) in Pará State, with particular reference to the role of *Psychodopygus wellcomei* as the vector of *Leishmania braziliensis braziliensis* in the Serra dos Carajás. Trans R Soc Trop Med Hyg..

[21] Freitas RA, Naiff RD, Barret TV (2002). Species diversity and flagellate infections in the sand fly fauna near Porto Grande, State of Amapá, Brazil (Diptera: Psychodidae. Kinetoplastida: Trypanosomatidae). Mem Inst Oswaldo Cruz..

[22] Lainson R, Shaw JJ, Peters W, Killick-Kendrick R (1987). Evolution, classification and geographical distribution. The Leishmaniases in Biology and Medicine.

[23] Galati EAB, Rangel EF, Shaw JJ (2018). Phlebotominae (Diptera, Psychodidae): classification, morphology, and terminology of adults and identification of American taxa. Brazilian Sand Flies: Biology, Taxonomy, Medical Importance and Control.

[24] Galati EAB, Galvis-Ovallos F, Lawyer P, Léger N, Depaquit J (2017). An illustrated guide for characters and terminology used in descriptions of Phlebotominae (Diptera, Psychodidae). Parasite.

[25] Afonso MMS, Duarte R, Miranda JC, Caranha L, Rangel EF. Studies on the feeding habits of *Lutzomyia* (*Lutzomyia*) *longipalpis* (Lutz & Neiva, 1912) (Diptera: Psychodidae: Phlebotominae) populations from endemic areas of American visceral leishmaniasis in Northeastern Brazil. J Trop Med. 2012;2012:858657. 10.1155/2012/858657 PMC3270439 22315621

[26] Brasil. Ministério da Saúde (2017). Secretaria de Vigilância em Saúde. Departamento de Vigilância das Doenças Transmissíveis.. Manual de Vigilância da Leishmaniose Tegumentar.

[27] Simon S, Veron V, Carme B (2010). *Leishmania* spp. identification by polymerase chain reaction-restriction fragment length polymorphism analysis and its applications in French Guiana. Diagn Microbiol Infect Dis..

[28] Floch H, Abonnenc E (1952). Diptères phlébotomes de la Guyane et des Antilles françaises. Faune de l’Union Française.

[29] Hammer Ø, Harper DAT, Ryan PD (2001). PAST: Paleontological Statistics Software Package for education and data analysis. Paleontol Electro..

[30] Léger N, Abonnenc E, Pajot F-X, Kramer R, Claustre J (1977). Liste commentée des phlébotomes de la Guyane française. Cahiers ORSTOM. Cahiers ORSTOM Ser Entomol Med Parasitol.

[31] Kocher A, Gantier J-C, Gaborit P, Zinger L, Holota H, Valiere S (2017). Vector soup: high-throughput identification of Neotropical phlebotomine sand flies using metabarcoding. Mol Ecol Resour.

[32] McDermott EG, Mullens BA (2018). The dark side of light traps. J Med Entomol.

[33] Souza AAA, Vasconcelos dos Santos T, YLL J, EAY I, Barata IR, MGS S (2016). Natural *Leishmania* (*Viannia*) spp. infections in phlebotomine sand flies (Diptera: Psychodidae) from the Brazilian Amazon region reveal new putative transmission cycles of American cutaneous leishmaniasis. Parasite.

[34] Rotureau B, Gaborit P, Issaly J, Carinci R, Fouque F, Carme B (2006). Diversity and ecology of sand flies (Diptera: Psychodidae: Phlebotominae) in coastal French Guiana. Am J Trop Med Hyg..

[35] Fouque F, Gaborit P, Issaly J, Carinci R, Gantier J-C, Ravel C, Dedet J-P (2007). Phlebotomine sand flies (Diptera: Psychodidae) associated with changing patterns in the transmission of the human cutaneous leishmaniasis in French Guiana. Mem Inst Oswaldo Cruz..

[36] Furtado NVR, Galardo AKR, Galardo CD, Firmino VC, Vasconcelos dos Santos T (2016). Phlebotomines (Diptera: Psychodidae) in a hydroelectric system affected area from northern Amazonian Brazil: further insights into the effects of environmental changes on vector ecology. J Trop Med.

[37] Forattini OP (1959). Sobre os flebótomos do território do Amapá, Brasil. Arq Fac Hig Saude Publica Univ Sao Paulo..

[38] Young DG, Duncan MA (1994). Guide to the identification and geographic distribution of *Lutzomyia* sand flies in Mexico, the West Indies, Central and South America (Diptera: Psychodidae). Gainesville. Mem Am Entomol Inst.

[39] Brazil RP, Andrade Filho JD, Falcão AL (2000). Notes on Phlebotomine sand flies (Diptera; Psychodidae) from Amapá state, Brazil. J Am Mosq Control Assoc.

[40] Oliveira AG, Andrade Filho JD, Falcão AL, Brazil RP (2001). A new sand fly, *Lutzomyia campograndensis* sp. n. (Diptera: Psychodidae: Phlebotominae) from the State of Mato Grosso do Sul, Brazil. Mem Inst Oswaldo Cruz..

[41] Saraiva JF, Souto RNP, Ferreira RMA (2011). Flebotomíneos (Diptera: Psychodidae) coletados em um assentamento rural no Estado do Amapá, Brasil. Biota Amaz.

[42] Galardo AKR, Galardo CD, Santana AA, Mendes JCC, Souza FRA, Duarte JP (2013). Primeira ocorrência de *Lutzomyia* (*Lutzomyia*) *longipalpis* Lutz & Neiva, 1912 (Diptera: Psychodidae: Phlebotominae) no Estado do Amapá, Brasil. Biota Amaz.

[43] Sábio PB, Andrade AJ, Galati EAB (2014). Assessment of the taxonomic status of some species included in the shannoni complex, with the description of a new species of *Psathyromyia* (Diptera: Psychodidae: Phlebotominae). J Med Entomol.

[44] Costa TS (2015). Identificação molecular (DNA barcode) de flebotomíneos (Diptera: Psychodidae: Phlebotominae) na Terra Indígena Wajãpi, Amazônia Oriental, Brasil. 2015. 94 f. Dissertation, Master in Biodiversidade Tropical.

[45] Aguiar GM, Vieira VR, Rangel EF, Shaw JJ (2018). Regional distribution and habitats of Brazilian phlebotomine species. Brazilian Sand Flies: Biology, Taxonomy, Medical Importance and Control.

[46] Pessoa FAC, Medeiros JF, Barret TV (2007). Effects of timber harvest on phlebotomine sand flies (Diptera: Psychodidae) in a production forest: abundance of species on tree trunks and prevalence of trypanosomatids. Mem Inst Oswaldo Cruz..

[47] Rosário IN, Andrade AJ, Ligeiro R, Ishak R, Silva IM (2017). Evaluating the adaptation process of sand fly fauna to anthropized environments in a leishmaniasis transmission area in the Brazilian Amazon. J Med Entomol..

[48] Ramos WR, Medeiros JF, Julião GR, Ríos-Velásquez CM, Marialva EF, Desmouliére SJM (2014). Anthropic effects on sand fly (Diptera: Psychodidae) abundance and diversity in an Amazonian rural settlement, Brazil. Acta Trop.

[49] Pereira Filho AA, Bandeira Mda C, Fonteles RS, Moraes JL, Lopes CR, Melo MN, Rebêlo JM (2015). An ecological study of sand flies (Diptera: Psychodidae) in the vicinity of Lençóis Maranhenses National Park, Maranhão, Brazil. Parasit Vectors.

[50] Souza NA, Andrade-Coelho CA, Silva VC, Peixoto AA, Rangel EF (2005). Moonlight and blood-feeding behaviour of *Lutzomyia intermedia* and *Lutzomyia whitmani* (Diptera: Psychodidae: Phlebotominae), vectors of American cutaneous leishmaniasis in Brazil. Mem Inst Oswaldo Cruz.

[51] Mellor HE, Hamilton JGC (2003). Navigation of *Lutzomyia longipalpis* (Diptera: Psychodidae) under dusk or starlight conditions. Bull Ent Res.

[52] Fernández MS, Santini MS, Diaz JI, Villarquide L, Lestani E, Achinelly M (2016). Parasitism by tylenchid nematodes in natural populations of *Pintomyia fischeri* (Diptera: Psychodidae: Phlebotominae) in Argentina. SM Trop Med J.

[53] Lantova L, Volf P. Mosquito and sand fly gregarines of the genus *Ascogregarina* and *Psychodiella* (Apicomplexa: Eugregarinorida, Aseptatorina) - overview of their taxonomy, life cycle, host specificity and pathogenicity. Infect Genet Evol. 2014;28:616–27. 10.1016/j.meegid.2014.04.021 24797386

[54] Lainson R, Shaw JJ, Póvoa MM (1981). The importance of edentates (sloths and anteaters) as primary reservoirs of *Leishmania braziliensis guyanensis*, causative agent of ‘pian bois’ in north Brazil. Trans R Soc Trop Med Hyg.

[55] Roque AL, Jansen AM (2014). Wild and synanthropic reservoirs of *Leishmania* species in the Americas. Int J Parasitol Parasites Wildl.

[56] Le Pont F, Pajot FX, Reguer R (1980). Preliminary observations on the silvatic cycle of leishmaniasis in French Guiana. Trans R Soc Trop Med Hyg.

[57] Pinheiro FG, Luz SLB, Franco AMR (2008). Infecção natural por tripanosomatídeos (Kinetoplastida: Trypanosomatidae) em *Lutzomyia umbratilis* (Diptera: Psychodidae) em áreas de leishmaniose tegumentar americana no Amazonas, Brasil. Acta Amaz.

[58] Le Pont F, Pajot FX (1980). La leishmaniose en Guyane Française. I. Étude de l’écologie et du taux d’infection naturelle d’un vecteur *Lutzomyia* (*Nyssomyia*) *umbratilis* Ward et Fraiha, 1977 en saison sèche. Considerations epidémiologiques. Cahiers ORSTOM Ser Entomol Med Parasitol.

[59] Brazil RP, Almeida DC, Brazil BG, Mamede SM (1991). Chicken house as a resting site of sand flies in Rio de Janeiro, Brazil. Parassitologia.

[60] Teodoro U, La Salvia FV, Lima EM, Spinosa RP, Barbosa OC, Ferreira MEMC (1993). Observações sobre o comportamento de flebotomíneos em ecótopos florestais e extraflorestais, em área endêmica de leishmaniose tegumentar americana, no norte do Estado do Paraná, sul do Brasil. Rev Saude Publica..

[61] Dias FDOP, Lorosa ES, Rebêlo JMM (2003). Fonte alimentar sangüínea e a peridomiciliação de *Lutzomyia longipalpis* (Lutz & Neiva, 1912) (Psychodidae, Phlebotominae). Cad Saude Publica.

[62] Ávila MM, Brilhante AF, de Souza CF, Bevilacqua PD, Galati EAB, Brazil RP (2018). Ecology, feeding and natural infection by *Leishmania* spp. of phlebotomine sand flies in an area of high incidence of American tegumentary leishmaniasis in the municipality of Rio Branco, Acre, Brazil. Parasit Vectors.

[63] Sant’Anna MRV, Nascimento A, Alexander B, Dilger E, Cavalcante RR, Diaz-Albiter HM (2010). Chicken blood provides a suitable meal for the sand fly *Lutzomyia longipalpis* and does not inhibit *Leishmania* development in the gut. Parasit Vectors.

[64] Christensen HA, Arias JR, Vasquez AM, Freitas RA (1982). Hosts of sand fly vectors of *Leishmania braziliensis guyanensis* in the central Amazon of Brazil. Am J Trop Med Hyg.

[65] Kocher A, de Thoisy B, Catzeflis F, Valière S, Bañuls AL, Murienne J (2017). iDNA screening: disease vectors as vertebrate samplers. Mol Ecol.

[66] Nery LCR, Lorosa ES, Franco AMR (2004). Feeding preference of the sand flies *Lutzomyia umbratilis* and *L. spathotrichia* (Diptera: Psychodidae, Phlebotominae) in an urban forest patch in the city of Manaus, Amazonas, Brazil. Mem Inst Oswaldo Cruz.

[67] Ferreira RC, Souza AA, Freitas R, Campaner M, Takata CSA, Barret TV, Shaw JJ (2008). Phylogenetic lineage of closely related trypanosomes (Trypanosomatidae, Kinetoplastida) of anurans and sand flies (Psychodidae, Diptera) sharing the same ecotopes in Brazilian Amazonia. J Eukaryot Microbiol.

[68] Kent A, Santos TV, Gandadin A, Samjhawan A, Mans DRA, Schallig HDFH (2013). Studies on the sand fly fauna (Diptera: Psychodidae) in high-transmission areas of cutaneous leishmaniasis in the Republic of Suriname. Parasit Vectors.

[69] Ready PD, Lainson R, Shaw JJ (1983). Leishmaniasis in Brazil: XX. Prevalence of ‘enzootic rodent leishmaniasis’ (*Leishmania mexicana amazonensis*), and apparent absence of ‘pian bois’ (*Le. braziliensis guyanensis*), in plantations of introduced tree species and in other non-climax forests in eastern Amazonia. Trans R Soc Trop Med Hyg.

